# Grasping Kinematics from the Perspective of the Individual Digits: A Modelling Study

**DOI:** 10.1371/journal.pone.0033150

**Published:** 2012-03-07

**Authors:** Rebekka Verheij, Eli Brenner, Jeroen B. J. Smeets

**Affiliations:** Faculty of Human Movement Sciences, Research Institute MOVE, VU University, Amsterdam, The Netherlands; The University of Western Ontario, Canada

## Abstract

Grasping is a prototype of human motor coordination. Nevertheless, it is not known what determines the typical movement patterns of grasping. One way to approach this issue is by building models. We developed a model based on the movements of the individual digits. In our model the following objectives were taken into account for each digit: move smoothly to the preselected goal position on the object without hitting other surfaces, arrive at about the same time as the other digit and never move too far from the other digit. These objectives were implemented by regarding the tips of the digits as point masses with a spring between them, each attracted to its goal position and repelled from objects' surfaces. Their movements were damped. Using a single set of parameters, our model can reproduce a wider variety of experimental findings than any previous model of grasping. Apart from reproducing known effects (even the angles under which digits approach trapezoidal objects' surfaces, which no other model can explain), our model predicted that the increase in maximum grip aperture with object size should be greater for blocks than for cylinders. A survey of the literature shows that this is indeed how humans behave. The model can also adequately predict how single digit pointing movements are made. This supports the idea that grasping kinematics follow from the movements of the individual digits.

## Introduction

Human grasping kinematics have certain characteristics. For example, there is a characteristic relation between the maximum distance between the tips of the digits on their way to an object and the size of the object that is to be grasped. Various experiments have been done and models have been made to test specific ideas about why precisely these kinematics emerge. In this study we will focus on the modeling part.

One popular idea, originally suggested by Jeannerod [1], is that the kinematics emerge from transporting the hand (i.e. wrist) toward the target (transport component) and at the same time opening and closing the hand in accordance with the dimensions of the object (grip component). A two-dimensional model based on this idea is presented by Hoff and Arbib [2]. This model consists of three motor activity generators: one for transport of the hand, a second one to preshape the hand and a third one to close the hand at the end of the movement. For the transport generator the constraints are on the hand (the start and end position, velocity and acceleration of the hand). For the preshape generator the constraints are on the aperture (initial and final aperture and aperture velocity). For the enclose generator the constraint is object size. The transport, preshape and enclose generators are coordinated in time. The movement time is preset and is an input for the transport generator. The model has at least 10 parameters that do not follow unambiguously from the constraints.

An alternative view, suggested by Smeets and Brenner, is that the kinematics of the grasping movement follows from the movements of the individual digits [3]. They presented a very simple two-dimensional minimum-jerk model in which the kinematics were determined by the constraints on the individual digits at the start and end of the movement. As in the model of Hoff and Arbib, movement time is preset. In addition to the movement time, the model has only one parameter that does not follow from the constraints: the approach parameter.

A limitation of both models, together with most other kinematic models [2]–[9], is that they all (deliberately) only consider movements in an environment without obstacles. A category of models that can deal with obstacles is models for which the outcome is based on evaluating remembered postures before movement onset (e.g. [10]–[12]). However, this is hard to reconcile with the capability of humans to make short latency online corrections during their grasping movements [13]–[15]. Moreover, such models can be used to simulate obstacle avoidance but they cannot predict behavior in new situations in a reproducible manner because a set of experienced postures is an input for the model.

In order to better test the origins of grasping kinematics we developed a model that is able to deal with obstacles and online corrections. We based our model on the view of Smeets and Brenner [3] that kinematics of grasping movements follow from moving each digit to its goal position. When building a model one can impose constraints that are fulfilled by definition. It is also possible to implement objectives for which the extent to which they influence the movement depends on the situation. The minimum jerk model is only based on constraints. This makes it virtually impossible to model objectives such as avoiding collisions with other digits or obstacles. We therefore chose to build a new model that combines task constraints with objectives such as to avoid collisions.

Models are a simplification of reality. In general we consider the simplest model that can describe the data to be the best. Such a model will include all essential components for a realistic outcome, and neglect all non-essential components. When looking at grasping kinematics, this raises the question whether it is essential to consider the human anatomy [11], [16]. Experimental studies have shown that trajectories of end-effectors are largely independent of the underlying joint movements [17]–[21]. For example, grasping kinematics are very similar when we grasp with digits of two hands instead of with two digits of one hand [22], [23]. We therefore only consider anatomy where we expect it to restrict the movements of the end-effectors. In the model of Smeets and Brenner [3] the physical link between the digits was not considered (but note that in that paper the movement times of the thumb and index finger were forced to be equal). In our model we consider the physical link between the digits because it is obvious that the movements of the digits are restricted with respect to each other to some extent. We are aware that the choice to neglect all further anatomy will lead to unrealistic predictions in extreme conditions where other restrictions imposed by anatomy become important. We accept this limitation. As is common for grasping research, the end-effectors we study are the tips of the index finger and the thumb. In the remainder of this article we will call these points ‘tips’.

The following task constraints were implemented for each tip: the initial position, the initial velocity, the initial acceleration and the goal position. Next to the task constraints, the following objectives were implemented for each tip: arrive at the goal position at about the same time as the other digit, move not too far from the other digit, move smoothly and do not hit the table, obstacles, other parts of the target object or the other digit. We chose to implement the objectives by a force field. We modeled the tips as point masses. The grasping kinematics then follow from the second law of Newton: the sum of the forces is mass times acceleration (see ‘model equations’ for more details).

The force field consists of four forces. The objective to arrive at the preselected goal position was implemented by a force *F_a_*, which attracts the tip toward its goal position. Next to this, there are repulsive forces perpendicular to the surface of each object. The sum of these repulsive forces, *F_r_*, represents the objective to avoid collision with positions other than the goal position. The target object and obstacles are treated in the same manner, only the table is considered a special type of obstacle with its own repulsive force. There is also a force of a virtual spring between the two tips, *F_s_*. This virtual spring prevents unintentional collisions between the tips and at the same time limits the distance between the tips. Smooth movements and the simultaneous arrival of both tips were implemented by a fourth (damping) force, *F_d_*, which is directed in the opposite direction to the velocity of the tip.

This model based on the forces *F_a_*, *F_r_*, *F_s_* and *F_d_*, has a close similarity with the potential field methods that are quite popular in on-line collision avoidance for robot manipulators. In these methods, the robot follows the gradient of a potential field consisting of attractive potentials due to the goal positions, repulsive potentials due to obstacles, and repulsive potentials due to joint limits [24]. Although virtual forces are used to calculate the trajectories of the tips, our model gives no predictions regarding forces exerted by the digits (let alone the muscles).

The overall aim of this study was to test the credibility of the view that grasping kinematics are determined by the movements of the individual digits to their goal positions. Based on a comparison of experimental outcomes with model predictions this view indeed seems credible.

## Results

In the following paragraphs, we provide an overview of how the model reproduces some commonly reported characteristics of grasping movements. We start by choosing values for each of our six model parameters that make our model reproduce the classical results of Jeannerod (outlined under ‘grasping a rod’) [1]. Subsequently, we test whether the model predicts grasps for objects of different sizes correctly. After that, we discuss a new prediction (concerning maximum grip aperture (MGA) and object shape). Next, we examine the effects of target distance, and then turn to grasping in the proximity of obstacles, online corrections and special cases (grasping with varying initial aperture and grasping trapezoidal targets). In addition, we show that the model is capable of producing realistic pointing movements.

The results of our model are not very sensitive to the exact choice of the parameter values, but they are sensitive enough to be able to generate different movements for different circumstances (see sensitivity analysis in methods section). Varying the values could be used to simulate differences between subjects and between trials, and to fit the model to data for specific circumstances. We choose to keep the parameters constant for all simulations in the results section to show that the observed features arise from our approach (formulating constraints and objectives for the digits and converting them to movements) rather than from fitting appropriate parameters. Thus, we do not claim that the selected values for the parameters are the best for reproducing any of the data that we consider. Moreover, we expect that the measures that they represent do actually differ across conditions and experiments. The comparison of our model predictions to experimental findings will therefore be largely qualitative.

### Commonly reported grasping characteristics

#### Grasping a rod

In order to choose values for our model parameters, we simulated one of the grasping movements studied by Jeannerod [1]: a grasping movement to a 10 cm high rod with a diameter of 2 mm placed on a table 40 cm in front of the digits. In the simulations we placed the goal positions at a height of 5 cm on the rod and at an angle of 30 degrees in the horizontal plane (where 0 degrees is a final grip orthogonal to the direction from the starting point to the object's centre). The table was modeled as a horizontal surface (60 cm×30 cm).

**Figure 1:**
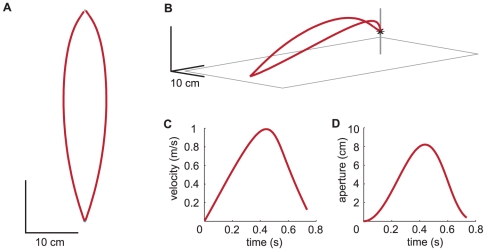
Example set of trajectories generated by the model. A: Paths of the tips in the horizontal plane. B: A perspective plot of the tips' paths and of the rod on the table. C: Mean velocity profile of both tips. D: Time-course of grip aperture.

The resulting position and velocity profiles of the tips are depicted in Fig. 1. The tips move higher than the final grasping points and then descend to grasp the rod. The velocity profile is approximately bell-shaped. Maximum velocity (MV) and MGA occur at the same time. MV is 0.99 m/s. The movement time (MT) is 0.73 s. This is in accordance with the findings of Jeannerod, who found that the digits move higher than the final grasping points and then descend to grasp the rod with an approximately bell-shaped velocity profile. In Jeannerod's study MT varied across subjects. Mean MT ranged between 0.72 s in the fastest subject and 1.18 s in the slowest. MV varied between 0.80 m/s and 1.35 m/s. The time difference between MV and MGA was 80 ms or less for all subjects [1]. Note that the fact that the model matched these experimental observations is not a coincidence, because the values of the parameters (table 1) were chosen to achieve this.

**Table 1:** Values chosen for the parameters of the model.

Parameter	Value
*A*	1.00 m^2^ s^−2^
*R_o_*	0.60 m^2^ s^−1^
*R_t_*	3⋅10^−2^ m^2^ s^−1^
*K*	13.0 s^−2^
*E*	0.07 m
*D*	0.16 m

#### Varying target size and shape

The best-studied aspect of grasping is the relationship between target size and grip aperture. We therefore start by examining effects of target size on our model's behavior. We modeled grasping movements to either a 10 cm high cylinder or a 10 cm high block on a horizontal surface (60 cm×30 cm). The target object was 40 cm from the hand. The cylinder's diameter and the width of the block could be 2, 3, 4, 5, 6, 7 or 8 cm. For every diameter of the cylinder we performed two simulations, one in which the cylinder was grasped at an angle of 30 degrees in the horizontal plane and one in which the cylinder was grasped at an angle of 0 degrees. For every width of the block we performed one simulation in which the block was grasped under an angle of 0 degrees in the horizontal plane. The block's depth was always equal to its width.

**Figure 2:**
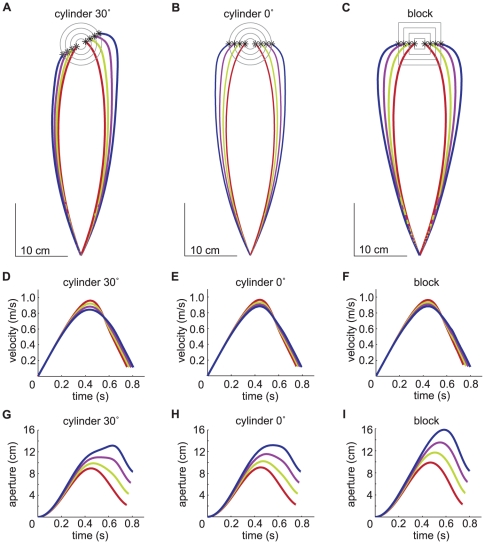
Simulating grasping movements to target objects of various sizes. A: Paths of the tips when grasping a cylinder at an angle of 30 degrees. B: Paths of the tips when grasping a cylinder at an angle of 0 degrees. C: Paths of the tips when grasping a block. D: Mean velocity profiles of both tips when grasping a cylinder at an angle of 30 degrees. E: Mean velocity profiles of both tips when grasping a cylinder at an angle of 0 degrees. F: Mean velocity profiles of both tips when grasping a block. G: Aperture profiles when grasping a cylinder at an angle of 30 degrees. H: Aperture profiles when grasping a cylinder at an angle of 0 degrees. I: Aperture profiles when grasping a block.

The resulting position, velocity and aperture profiles are depicted in Fig. 2. For cylinders, the shape of the aperture profile varies across object sizes (Fig. 2G,H). Aperture profiles with plateaus, as we see in our simulations of grasps to larger cylinders, have been reported by Bongers and colleagues [25].

**Figure 3:**
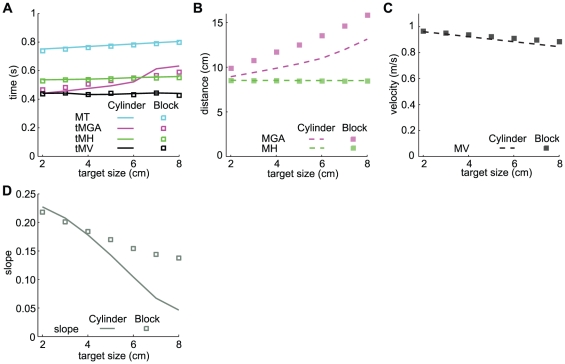
The dependence of kinematic parameters of grasping on target size and shape. A: Movement time (MT), time to maximum grip aperture (tMGA), time to maximum height (tMH) and time to maximum velocity (tMV). B: Maximum grip aperture (MGA) and maximum height (MH). C: Maximum velocity (MV). D: The slope of the relationship between MGA and target distance (Figure 5B) decreases with target size.

The values for several measures predicted by our model for grasping a cylinder at an angle of 30 degrees and for grasping a block are given in Fig. 3. For the maximum height of the hand, most authors report the height of the wrist marker. Since our model only covers the trajectories of the tips, we took the mean height of the two tips as a measure for height. When grasping a cylinder, the absolute time to MGA (tMGA) changes rapidly between target sizes of 6 and 7 cm. This is because the aperture profile has a broad peak and the maximum value shifts from the left to the right side of this peak between the target sizes of 6 and 7 cm (Fig. 2G). The model predicts a slight increase in MT with target size. In the literature an increase in MT [23], [26], [27], no effect on MT [28]–[31], and a decrease in MT [32]–[34] with target size have all been found. For tMGA our model predicts an increase with target size, which is in accordance with the literature [29], [31], [32], [34], [35]. The model predicts negligible effects of target size on the absolute time to maximum velocity (tMV) and the absolute time to maximum height (tMH). For tMV no effect of target size was found experimentally [29], [34]. We could not find an experimental study describing the effect of target size on tMH. The model predicts a strong increase of MGA with target size. We will discuss the effect of size on MGA in detail in the following paragraphs. The model predicts no effect of target size on maximum height (MH), while experimentally a slight increase (at most 1.5 mm height per 1 cm of target size) is found [35], [36]. A slight decrease of MV with target size is predicted. In the literature no effect of target size on MV [29], [30], [32], [34] or an increase of MV [27], [35] is found.

For most measures, the shape of the object does not seem to influence the value much. However, according to the model, MGA and the increase of MGA with target size are larger for the block than for the cylinder (Fig. 3B). To examine whether this is also true for real grasping we used experimental data of several studies. We only included studies in which the target and hand were continuously visible with both eyes, the target was stationary and the subjects were healthy adults. 12 studies in which a cylinder was grasped [22], [29]–[31], [34], [37]–[43] and 6 studies in which a block was grasped [23], [28], [32], [44]–[46] fulfilled the inclusion criteria. In 10 studies in which a cylinder was grasped [47]–[56] and in 10 studies in which a block was grasped [26], [27], [33], [35], [57]–[62] only a subgroup of the measurements conformed to these inclusion criteria, so we took the data of that subgroup. Thus, altogether we used the data of 22 studies in which a cylinder was grasped and 16 studies in which a block was grasped.

To evaluate the prediction that the MGA is larger for the block than for the cylinder we calculated the mean MGA for targets with a size of 5 cm over all selected studies. When there was no target object with a size of 5 cm we interpolated or extrapolated the data. The mean MGA for studies in which a block was grasped is 9.4 cm (SD = 1.7 cm) and the mean MGA for studies in which a cylinder was grasped is 8.7 cm (SD = 1.4 cm). The difference in MGA was in the predicted direction, but not significant (t(36) = −1.31, p = 0.20; two-tailed independent samples t-test). One problem with comparing MGA across studies is that the marker placement differs across studies, which adds a lot of variability. A more reliable measure is therefore the change in MGA with target size (Fig. 6 in [3]).

**Figure 4:**
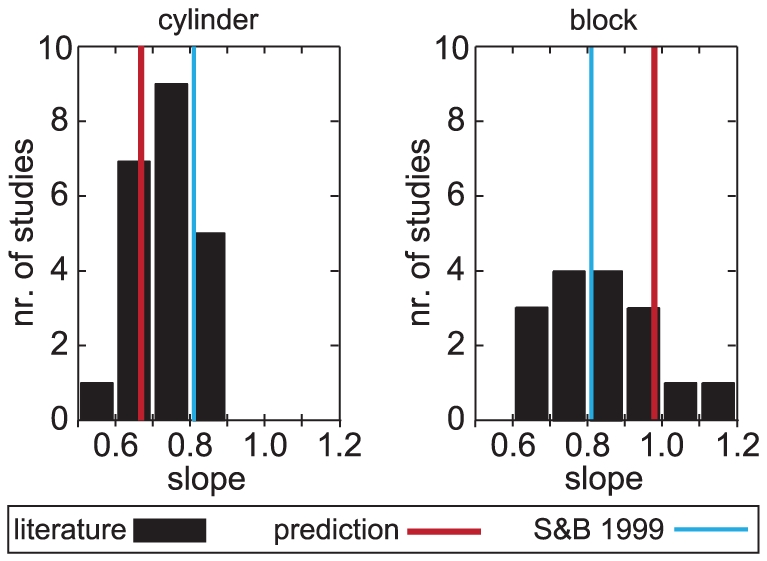
Distribution of the slopes of linear fits to the relationship between MGA and target size. The slopes are taken from the data of 38 published studies (see text for selection). The red lines represent the values of the slopes predicted for the two object shapes by our model and the blue lines represent the single value predicted by the model of Smeets and Brenner.

To evaluate the prediction that the increase in MGA with target size is larger for the block than for the cylinder we performed linear regressions for MGA as a function of target size. For our model we obtained a value of 0.67 for the slope when the target was a cylinder grasped under an angle of 30 degrees and a value of 0.68 when the cylinder was grasped under an angle of 0 degrees. When the target was a block we obtained a value of 0.98 for the slope. In line with the model prediction, linear regression for MGA as a function of object size yielded larger slopes for the studies with a block (mean = 0.83, SD = 0.14) than for the studies with a cylinder (mean = 0.73, SD = 0.09), see Fig. 4. The difference in slope was significant (t(36) = −2.59, p = 0.014; two-tailed independent samples t-test). Object shape does not influence the increase of MGA with target size in the model of Smeets and Brenner [3].

According to our model, the relative time to MGA increases with increasing target size: from 59% to 79% for the cylinder and from 63% to 74% for the block. Such an increase in the relative time to MGA with target size is in line with the literature. The predicted values are within the experimentally found range: from 55 to 85% for target sizes between 2 and 8 cm (reviewed in [3]).

#### Varying target distance

To examine the effect of target distance, we ran our model with a cylinder on a horizontal surface (60 cm×30 cm) at various distances from the tips. The cylinder was 10 cm high, had a diameter of 4 cm and was grasped at a height of 5 cm and at an angle of 30 degrees in the horizontal plane. Likewise, grasping movements were simulated to a virtual block with a width and depth of 4 cm and a height of 10 cm. The block was grasped at a height of 5 cm and under an angle of 0 degrees in the horizontal plane.

**Figure 5:**
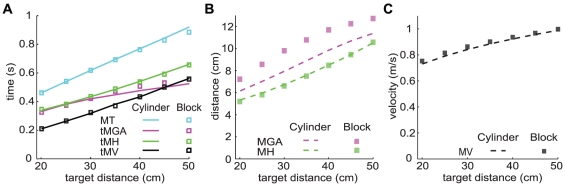
The dependence of kinematic parameters of grasping on target distance. The target was a cylinder or a block (width 4 cm). A: Movement time (MT), time to maximum grip aperture (tMGA), time to maximum height (tMH) and time to maximum velocity (tMV) all increase with target distance. B: Maximum grip aperture (MGA) and maximum height (MH) increase with target distance. C: Maximum velocity (MV) increases with target distance.

Experimental studies have found that MV [1], [36], [63]–[65], tMV [65], MH [36], [63], tMH [26], [66], tMGA [36], [63], [65], [67] and MT [36], [63], [65] all increase with target distance. Our model produces movements with these characteristics for both cylinders and blocks (Fig. 5).

Our model predicts an increase of MGA with target distance for both cylinders and blocks. Several studies found an increase in MGA with distance when grasping a cylinder [26], [36], [63], [67] or a block [33], [35], although the experiments included conditions without continues visual feedback. Chieffi and Gentilucci [51] found that the relation between MGA and target distance depends on the dimensions of the target. They found that MGA increased with target distance when the target diameter was between 1 and 4 cm. The increase was highest for the smallest target diameters. When the target diameter was 5 or 6 cm they did not find a significant influence of distance on MGA. These outcomes suggest that the slope of the relationship between MGA and distance decreases with target size and is zero for large targets. This is in line with the study of Jakobson and Goodale [35] who reported a stronger effect of distance on MGA for small blocks compared to large blocks.

To explore whether our model also predicts a decrease in slope with object size, we repeated the simulations of the effect of target distance on MGA for various sizes. For each target size we calculated the slope of the relation between MGA and target distance. In line with the literature [35], [51], our model predicts a decrease in slope with increasing target size (Fig. 3D). The model of Smeets and Brenner [3] predicts that MGA does not depend on the target distance.

#### Grasping in the proximity of obstacles

**Figure 6:**
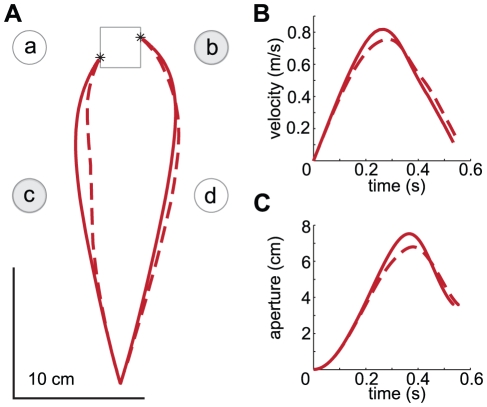
Simulating a grasping movement in the proximity of obstacles. Solid red lines represent the condition with no obstacles. Dashed red lines represent the condition with obstacles at ‘b’ and ‘c’. A: Paths of the tips in the horizontal plane. Circles indicate possible obstacle positions. B: Velocity profiles, averaged over both tips. C: Time-course of grip aperture.

To examine the effect of obstacles we simulated part of the experiment of Mon-Williams and colleagues [68] in which obstacles were placed near the target and near the path to the target. As in the experimental study, the target object was a block with sides of 3 cm and a height of 10 cm, placed on a table 25 cm in front of the digits. Obstacles were cylinders with a diameter of 2.5 cm and a height of 10 cm. Obstacles could be placed at the four positions shown in Fig. 6. We simulated trials with one obstacle placed at one of the positions ‘a’, ‘b’, ‘c’ or ‘d’, and trials with two obstacles placed at positions ‘a’ and ‘d’ or at positions ‘b’ and ‘c’. Based on Fig. 1 of the original study [68] the goal position of the thumb was chosen on the left side, 0.75 cm closer than the target center at a height of 5 cm. The goal position of the index finger was chosen on the right side, 0.75 cm further than the target center at a height of 5 cm.

The model predicts an increase of MT and a decrease of MV in all conditions in which a single obstacle is present. Experimentally, larger effects were found in the predicted direction (Table 2). In the experimental study it was observed that the increase of MT was smallest for position ‘a’. The model predicts the smallest increase for position ‘a’ as well. Experimentally, a decrease of MGA was found for all obstacle positions. The model predicts a decrease for all positions except position ‘a’.

**Table 2:** Predicted and experimentally found differences in MT, MGA and MV between movements without obstacles and movements with one or two obstacles.

		a	b	c	d	ad	bc
Δ MT (ms)	Model	9.0	10.5	9.5	11.5	21.2	20.2
	Exp.	23.5	47.4	43.4	55.0	59.01	77.2
Δ MGA (mm)	Model	1.0	−1.6	−5.7	−12.6	−11.8	−7.3
	Exp.	−10.9	−13.6	−9.3	−4.7	−15.3	−20.8
Δ MV (mm/s)	Model	−22.8	−29.8	−25.5	−62.2	−91.4	−62.0
	Exp.	−80.0	−118.0	−124.0	−128.0	−163.0	−204.0

Mon-Williams and colleagues compared the movements when two obstacles were present together at positions ‘a’ and ‘d’ with when there was one obstacle at ‘a’ or ‘d’. They found that MT, MGA and MV were affected to a greater extent when two obstacles were present. The same holds for a comparison of movements when two obstacles were present together at positions ‘b’ and ‘c’ with when there was one obstacle at ‘b’ or ‘c’. The model also predicts a larger effect on MT and MV when two obstacles are present instead of one. A larger effect on MGA was only predicted when obstacles were present at ‘b’ and ‘c’ compared to either at ‘b’ or at ‘c’.

Experimentally the effect on MT and MV is larger for condition ‘bc’ compared to condition ‘ad’. Since the environment is mirrored between these two conditions, an explanation might be based on the asymmetry in human anatomy. In condition ‘bc’ the fingers come much closer to obstacle ‘b’, than the thumb comes to obstacle ‘a’ in condition ‘ad’ (the fingers have to flex more than the thumb because of their length). Since the length of the digits is not included in our model, this finding is obviously not predicted.

Although the size of the obstacles' effects is smaller in the model prediction than is found experimentally, the direction of most effects correspond well. We could easily provide a better match with the experiment by changing the repulsiveness of the obstacles. This would not influence the outcomes of the simulations without obstacles if the repulsiveness of the target object and of the obstacles were set independently.

In the study discussed in the previous paragraphs [68], the obstacles were placed between the starting point and the target, near the hand's path, resulting in a decrease in MGA. Tresilian [69] placed an obstacle 4.5 cm behind the center of the target. He reported that the mean MGA increased slightly (for three out of four subjects) due to this obstacle. Averaged across subjects, the mean increase in MGA was 1.9 mm. The standard deviation was not reported. We simulated his experiment and also found a slight increase (1.4 mm) in MGA when the obstacle was placed behind the target. No other model would predict such an increase of MGA due to an object behind the target [2], [3], [10]–[12].

#### Online corrections

**Figure 7:**
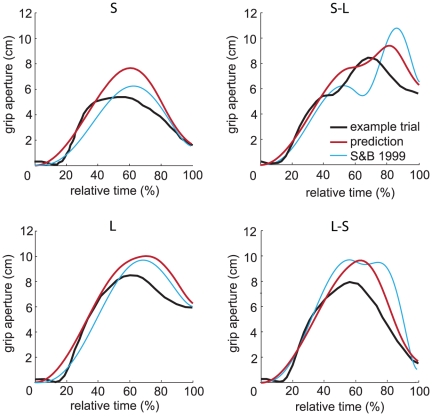
Grip aperture profiles during trials with constant and changing target diameters. The black lines indicate representative trials measured by Paulignan et al. (Fig. 8 of [71]). To remove the effect of marker placement in the experimental data, we shifted the emperical curves downwards so that the minimum aperture is 0 cm. The red lines represent the grip aperture profiles generated by our model and the blue lines represent the grip aperture profiles generated by the model of Smeets and Brenner (Fig. 3 of [70]). ‘S’ and ‘L’ indicate the conditions in which the diameter was constant, 1.5 cm or 6 cm respectively. ‘S-L’ indicates the condition in which the diameter changed from 1.5 cm to 6 cm and ‘L-S’ indicates the condition in which the diameter changed from 6 to 1.5 cm.

We consider the ability to deal with perturbations to be an essential aspect of a model of grasping kinematics [2], [70]. Paulignan et al. [71] examined prehension movements in which the diameter of a target cylinder changed at movement onset. They found that grip aperture was affected by the perturbation. When the target diameter increased from 1.5 cm to 6 cm they found two stages in the increase in grip aperture (an early peak around 40% of MT and a second peak around 70%, see Fig. 7). The height of the first peak corresponded to the MGA observed in control trials in which the target diameter was 1.5 cm and the height of the second peak corresponded to the MGA observed in control trials in which the target diameter was 6 cm. They report that the distinction between the two peaks was not as clear for all subjects.

We simulated grasping movements to cylinders with the same dimensions as those of the experiment of Paulignan et al. In two simulations the diameter was constant, either 1.5 cm or 6 cm and in two simulations the diameter changed, either from 1.5 cm to 6 cm or from 6 cm to 1.5 cm. The initial distance between the digits and the target was equal to the distance in their experiment (35 cm). The cylinders were grasped at an angle of 30 degrees in the horizontal plane. Humans respond to changes in the diameter with some latency. We obtained realistic trajectories if we changed the virtual target 400 ms after the perturbation.

As was found experimentally, our model shows two stages in the increase in grip aperture when the target diameter increased. The height of the initial peak corresponds to the MGA observed in the simulation in which the target diameter was 1.5 cm all the time. The height of the second peak corresponds to the MGA observed in the simulation in which the target diameter was 6 cm all the time. As was found experimentally, the two stages are present when the target size increased, but not when it decreased. The double peak pattern, in the condition in which the target size increased, becomes more pronounced when a larger value is taken for the reaction time and less pronounced when a smaller value, like 350 ms (used in [70]), is taken. We chose 400 ms because that results in similar profiles for the experimental data and the model outcome. Smeets and colleagues [70] simulated the same movement using the model of Smeets and Brenner [3] and found a double peak in the grip aperture when the target size increased as well as when the target size decreased.

### Special cases of grasping

In the following sections we further test our model by focusing on two special cases that few of the existing models have been able to incorporate.

#### Varying the initial aperture

**Figure 8:**
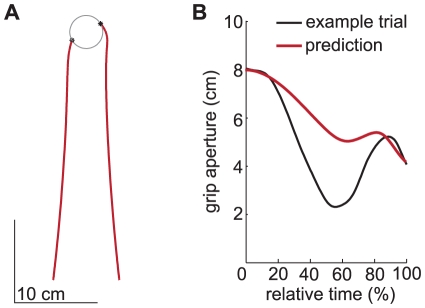
Results of simulating a grasping movement with a large initial aperture. The target was a 5.5 cm high cylinder with a diameter of 4 cm placed at a distance of 30 cm on a horizontal surface, as in the experiment by Hesse and Deubel [38]. The simulation started with the tips 8 cm apart. A: The position profiles of the tips when simulating grasping. B: The development of aperture in time. The black line represents a single trial of a representative subject measured by Hesse and Deubel (Fig. 3d of [38]). To remove the effect of marker placement in the experimental data, we shifted the emperical curve downwards so that the initial aperture is 8 cm. The red line represents the grip aperture profile given by our model.

It has been observed that when humans begin the reach-to-grasp movement with their thumb and index finger fully extended, the thumb and index finger initially start moving toward each other and grip then reopens before the object is grasped (paradoxical finger closure) [38], [40], [72], [73]. Most of the existing models [2], [4], [5], [7], [8], [10]–[12], [74] have been unable to reproduce paradoxical finger closure. An exception are neural network models [6], [9]. Our model reproduces this phenomenon. An example of a simulation in which paradoxical finger closure occurs is depicted in Fig. 8.

#### Grasping a trapezoid

**Figure 9:**
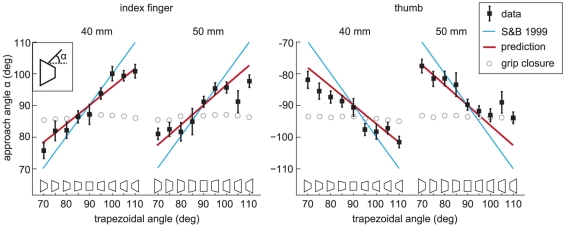
Approach angles of index finger and thumb when grasping trapezoid-shaped objects. Black solid squares indicate the experimentally found angles (α in inset, with the standard error across subjects), reproduced from Fig. 4 of [75]. The observed angles are more similar to the angles predicted by our model (red lines) than to the angles predicted by the model of Smeets and Brenner (blue lines) or to simple grip closure (grey open circles).

When Kleinholdermann et al. [75] examined the angles under which the digits approach trapezoidal objects' surfaces when grasped from above, they found that subjects tended to approach the objects' surfaces perpendicularly, but there was a clear deviation from the orthogonal approach predicted by Smeets and Brenner [3] (blue line in Fig. 9). If the grip had simply closed on the object, then the approach angle would not have changed with surface orientation (open circles in Fig. 9), since the digits would have approached the surface along the line connecting the contact positions. None of the existing models can explain their results.

We simulated grasping movements to trapezoidal objects with the same dimensions as those of the experiment of Kleinholdermann et al. The starting position of the tips was equal to the starting position in their experiment, 45 cm above the center of the target object. Our model predicts almost the same approach angles as were found experimentally (red lines in Fig. 9).

### Single-digit movements

**Figure 10:**
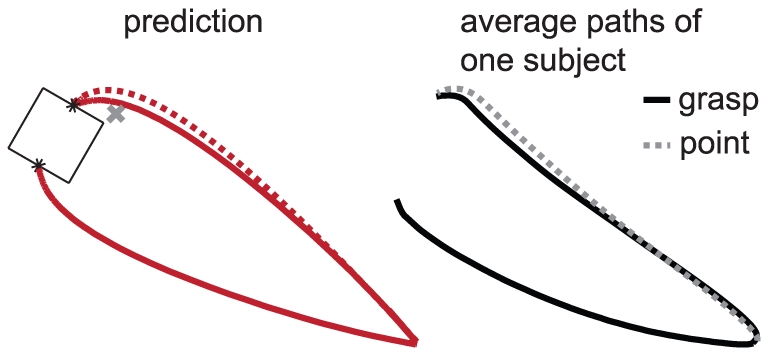
Paths of the tips in the horizontal plane for grasping and pointing. In both the simulation and the experimental study (Fig. 2 of [76]) the target was a cube (sides of 5 cm) placed at a distance of 30 cm from the digits. To simulate reaching to push on the side of the cube with the index finger, a realistic position was chosen as the goal position of the thumb (1 cm to the side of and 3 cm closer than the goal position of the index finger; indicated with a grey cross).

Although we designed our model for grasping, the implemented objectives are meaningful for single-digit movements too. The model should therefore be able to simulate single-digit pointing movements (again without changing the values of the parameters). The movement of a digit when reaching to push on the side of the object with that digit has been shown to be similar to its movement in a grasping task, but the digit moves slightly further to the side in the single-digit task [76]. In line with these results, our model predicts similar movements in grasping and pushing, with the digit moving slightly further to the side in the single-digit task (Fig. 10), supporting the view that grasping kinematics are dictated by the movements of the individual digits to their goal positions [3]. When the same approach parameter is used, the model of Smeets and Brenner predicts exactly the same movements for grasping and pointing.

## Discussion

We developed a model to help us gain more insight into the origin of grasping kinematics. We modeled the digits as point masses with a spring between them, each attracted to its goal position and repelled from objects' surfaces. The movement was damped. Each digit thus moves in a force field and the strength and direction of the force acting on the digit depends on the digit's velocity and position in the force field. Whereas the constraints in the model representing the view of Jeannerod were formulated for the hand position and grip aperture [2], in our model, like in the model of Smeets and Brenner [3], the task constraints are on the individual digits. The objectives to achieve a successful grasping movement were also implemented on the individual digits. Grip aperture is just one of the variables that determines what the force field working on each individual digit looks like.

Commonly reported grasping kinematics can be reproduced with our model. We used the same set of six parameters for all simulations, to make sure that the results are not due to fine-tuning of the parameters to each situation, although better fits could undoubtedly be achieved for each simulation individually. The sensitivity analysis described in the methods section shows that selected features of the movement can be controlled by changing the parameter values. Because the exact outcome will vary if we choose different values for the model parameters, we are primarily interested in the qualitative behavior of our model.

In general, the simulated paths and velocity profiles are similar to the experimental results. The simulations give values for MT, MV and MGA that are within the range of experimentally reported values. Most effects of increasing target distance or target size are in accordance with human movements. The model also proved to be able to deal with two special situations: grasping with a large grip aperture at the start of the movement and grasping trapezoidal targets.

Apart from reproducing known effects, our model predicted that the shape of the target object has an effect on the relation between object size and MGA. The increase in the MGA when the size of the target is increased is larger when the target is a rectangular block than when it is a cylinder. Comparing the results of various experimental studies revealed that this is indeed the case. As far as we know, no one has previously noticed this.

A nice result of the model is that it shows one could understand the (often neglected) vertical part of the grasping movement as a result of avoiding contact with the table during the movement. Whether this is a valid explanation awaits experimental verification.

A particular quality of our model is that it can simulate the small differences between the digits' movements in grasping and pointing, using the same equations and values for the parameters. In this way our model supports the view that grasping is in fact pointing with two fingers [3], [76].

One of the strengths of our model is the ability to deal with both obstacles and online corrections. Although the predicted extent to which obstacles influence the movement is smaller than has been found experimentally, most predictions are in the right direction. We chose to use the same parameter values for the repulsive force for the target and the obstacles. It is possible that humans find it more important to avoid hitting an obstacle than to avoid ending at a different position on the target than they initially selected. Based on this reasoning, we could have changed the strength of the repulsive force of the obstacles in order to get larger effects. It is likely that in real life collisions with different objects will not be considered equally undesirable. If the object is unstable or the potential cost of touching it is high, it is likely to have a “stronger repulsive force”.

Similarly, the model considers the geometry (shape and size) of the target, but we know that other properties like fragility [77] and surface roughness [78] affect the movement too. We assume that these other properties of the target influence the values of some of the model parameters. When grasping a fragile object, for example, one can imagine that the parameters are set to approach the target more slowly and accurately than when grasping non-fragile objects, leading to different trajectories for targets of the same shape. Variability in the parameter values would also give rise to variability in the movements, as is found both within and between subjects.

Our model assumes that people have full knowledge of the shape of all relevant surfaces. This is not likely, because people often grasp objects at positions that they cannot see, or move their hand behind obstacles. In such cases, one must imagine what the surface looks like. That people use such indirect information is evident. For example, subjects scale their hand opening to the target object's size even if they grasp it at points that they cannot see, which is often the case because the finger is frequently placed behind the target object. Moreover, scaling is found when people have to grasp a target that cannot be seen [35], [53].

The next issue we will consider is the angle with which the tip approaches the target surface. In the model of Smeets and Brenner [3] a perpendicular approach was one of the constraints. However, people do not always adopt a completely perpendicular approach [75]. In our model the objective to avoid hitting the target object at positions other than the goal position results in a tendency towards a perpendicular approach. The approach angle depends on the ratio between the repulsive force from the target surface and the attractive force to the goal position (as well as on factors such as scene layout). The larger the repulsive force compared to the attractive force, the more perpendicular the approach.

For simplicity, we chose to simulate only the movements of the tips of the index finger and thumb. In real-life we often grasp with five digits simultaneously. The technique described in this paper could be extended to simulate grasping with more than two digits. One way to do this is to include an extra point mass for every extra tip, connected to its neighboring tips by springs. The same kinds of forces act on these extra tips as on the tips representing the index finger and the thumb.

The last issue that we will consider is the relation between our model and how the central nervous system controls grasping. The aim of our modeling work was to show the mathematical feasibility of the view that task constraints and objectives for the individual digits determine grasping kinematics. Obviously we do not claim that the digits are point masses moving in a force field, this is just a convenient model with which it is possible to implement task constraints and objectives. We interpret the fact that task constraints and objectives can largely explain human grasping behavior as evidence that the neural and muscular substrates do not dominate the choice of solutions.

Why do we need a new model? Our model can account for more experimental findings than the model of Smeets and Brenner [3]. It can also account for the paths' curvature in the vertical plane, differences in the increase of MGA with target size for cylinders and blocks, paradoxical finger closure, decreased MGA and MV and increased MT when obstacles are placed in the proximity of the target, and that the digit moves slightly further to the side in pointing than in grasping. Moreover, it can be used to make predictions for movement times. However, the model of Smeets and Brenner is simpler and has fewer parameters. It is therefore much easier to use and thus preferable as a first modeling tool. Together the models increase the credibility of the view that grasping kinematics are determined by the task constraints for the individual digits [3].

## Methods

### Model equations

The total force acting on each tip is the sum of four forces, given by the equations below. Each force is a vector for which the direction is explained in the text. The equations only give the magnitude (scalars), of the forces. Kinematics follow from Newton's second law. For convenience, we express the forces per unit mass; the forces in the equations below thus have the dimension of acceleration.

**Figure 11:**
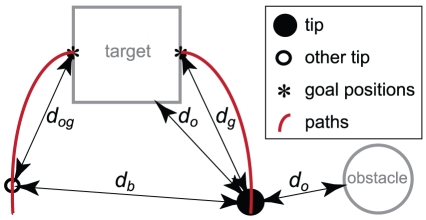
Distances used in the equations. The distances are indicated with respect to one of the tips. The distances to only one point on the surface of the target and obstacle (both indicated with *d_o_*) are shown; the model takes into account all relevant points and their respective distances (Fig. 12).

**Figure 12:**
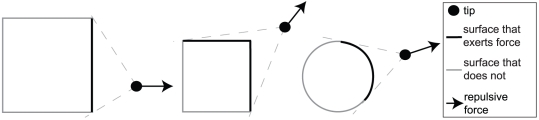
The repulsive force. Three examples to show the direction of the repulsive force and which surface areas exert a repulsive force on the tip.

The force that attracts the tip to the goal position, *F_a_*, is directed to the goal position and calculated according to:
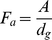
(1)The strength of the attractive force is set by the parameter *A*. *F_a_* increases when *d_g_*, the distance from the tip to the corresponding goal position, decreases. This is necessary since the target object is considered as an obstacle as well, and the repulsive force from an obstacle increases as the distance to the tip decreases (as will be explained later in this section). Fig. 11 illustrates *d_g_* and the distances used in the following equations. Each surface of an object that is visible from the position of the tip exerts a repulsive force on the tip. Three graphical examples are depicted in Fig. 12.

The total repulsive force exerted on the tip, *F_r_*, is calculated according to:
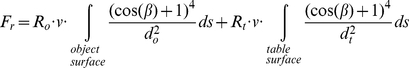
(2)It is the integral of the repulsive forces generated by the surface elements across the whole surface area that exerts a repulsive force on the tip. The repulsive forces are always orthogonal to their respective surface elements. *β*, *d_o_* and *d_t_* are functions of the position on the surfaces. The strength of the repulsive force from an object (target or obstacle) is set by the parameter *R_o_*, and the strength of the repulsive force from the table by the parameter *R_t_*. The repulsive force depends on the distance between the tip and the surface elements (d_o_ for the target and obstacles; d_t_ for the table) and on the angle *β* between the velocity of the tip and a line connecting the surface element with the tip. The more the movement direction is toward the element, the smaller is *β* and the larger the repulsive force should be to avoid a collision. By using cos(*β*)+1 we ensured that the repulsive force is highest when the movement is straight toward the element (*β* = 0) and zero when the movement is straight away from the element (*β* = π).

Next to this, *F_r_* increases when the velocity *v* of the tips increases. The reason for including the velocity in the equation is that human movements become more variable when the velocity increases [79], [80]. The larger the movement variability, the more objects should repel the tip in order to avoid a collision. Furthermore, when the movement is toward the obstacle, the urge to change the movement direction should increase with the velocity, simply because there is less time to change the movement direction if the digit is moving fast.

The two tips exert a force *F_s_* on each other. *F_s_* is equal but in opposite directions for the two tips so that the tips are attracted toward each other when they are far apart and pushed away from each other when close together. The magnitude of *F_s_* is calculated according to:
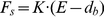
(3)
*F_s_* is thus modeled as a linear spring with stiffness *K* and equilibrium length *E*. The distance between the two tips is denoted by *d_b_*.

The force that damps the movement and help the tips arrive simultaneously, *F_d_*, is given by:
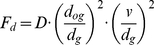
(4)F_d_ is directed in the opposite direction to the velocity of the tip. The strength can be set by the parameter *D*. *F_d_* increases when the velocity *v* of the tip increases, which is characteristic for a damper. *d_g_* is the distance between the tip for which *F_d_* is calculated, and its goal position. Near the goal position, the damping should increase rapidly in order to avoid hitting the surface with a high velocity. To achieve this the damping force contains the term 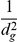.

To help ensure that the two tips arrive at about the same time, *F_d_* also depends on the ratio of the distances between the tips and their goal positions: *d_g_* for the tip for which the force is calculated and *d_og_* for the other tip. If the tip is closer to its goal position than the other tip (*d_g_*<*d_og_*), *F_d_* is larger so the tip slows down. In human grasping the digits do not always arrive at exactly the same time. Biegstraaten et al. [81] found that the index finger contacted the target on average 5 ms earlier than the thumb when the starting position was in front of the target. A small asymmetry is not inconsistent with the objective of simultaneous arrival, as implemented in our model. When a cylinder with a diameter of 4 cm and a height of 10 cm, placed at a distance of 40 cm, is grasped under an angle of 30 degrees, our model predicts that the index finger will arrive 2 ms earlier than the thumb.

Some arbitrary choices concerning the exponents of the individual terms were made in the four equations given above. We chose the current values by examining which combination of exponents and values of the six parameters produced movements that were similar to natural movements [1]. All simulations (except for the sensitivity analysis described below) were performed with the chosen set of parameters. The values are given in Table 1.

The grasping kinematics follow from the second law of Newton: the sum of the forces is mass times acceleration. Since the forces depend on the positions of the tips, this leaves us with a set of differential equations. Differential equations are equations in which an unknown function and one or more of its derivatives are present. In our case the unknown function is the position function. The highest derivative of the position function present in our equations is the second derivative, the acceleration. A second order differential equation was formed for each of the three directions of three-dimensional space for each tip. The system of six differential equations was integrated at once using the built-in MATLAB solver ode45. Except when we simulated the effect of a large initial aperture, the initial positions of the tips were always set 0.1 mm to the side of each other. Except when we simulated grasping movements to a trapezoid, the initial height of the tips above the table was always 1 mm. The initial velocity was always zero. We considered the moment at which the thumb was at a horizontal distance of 2 mm from its goal position to be the end of the movement.

### Sensitivity analysis

If our model resembles the way in which humans control their movements, we expect the values of the parameters to vary between people and across situations. If a parameter value must be varied to achieve some goal, such as moving faster, the behavior of the whole system may change, making the choice of parameters to achieve that goal a complex issue. We therefore determined how sensitive the model predictions are to the values of the parameters.

We simulated a grasping movement to a 10 cm high cylinder, 40 cm from the tips. The cylinder was grasped at an angle of 30 degrees in the horizontal plane at 5 cm height. The simulation was performed for two diameters of the cylinder: 2 cm and 6 cm.

The effect of decreasing and increasing the parameter values by 10% (with respect to the values given in Table 1) was examined separately for each model parameter. We considered the effect on MV, tMV, MGA, tMGA, MH, tMH and MT. Half the difference between the outcome for a 10% increase and a 10% decrease of each parameter was expressed as a percentage of the original value. These values are given for both cylinder sizes (Table 3).

**Table 3:** The effect of changing model parameter values on various measures.

	*A*		*R_o_*		*R_t_*		*K*		*E*		*D*	
Size (cm)	2	6	2	6	2	6	2	6	2	6	2	6
MV	**5.15**	**5.30**	−0.30	−0.84	0.08	0.17	−0.03	0.03	−0.04	0.11	−2.67	−2.30
tMV	**−5.27**	**−6.38**	0.10	0.11	0.32	0.34	−0.68	0.11	−0.61	0.03	−0.90	−1.64
MGA	−4.58	−2.00	0.11	0.91	−0.06	−0.05	4.53	1.50	**9.22**	**6.77**	−0.78	−0.86
tMGA	−3.38	2.20	2.30	2.31	−1.88	0.03	−2.84	**−8.37**	1.63	**−8.75**	−1.19	0.15
MH	−1.99	−2.05	0.06	0.35	3.63	3.52	0.18	0.06	0.18	0.00	−1.17	−1.00
tMH	**−5.23**	**−5.31**	0.09	0.42	0.39	−0.54	−0.15	−0.11	−0.07	0.14	0.74	0.56
MT	**−5.38**	**−5.49**	0.29	0.74	0.27	0.25	0.07	−0.06	0.31	0.19	2.08	1.95

Half the difference in kinematic variable (MV, tMV, MGA, tMGA, MH, tMH and MT) between the outcome for a 10% increase and a 10% decrease of each model parameter (A, R_o_, R_t_, K, E and D) expressed as a percentage of the original value of the kinematic variable. Results are shown for cylinders with diameters (sizes) of 2 cm and 6 cm. The outcomes that change by more than 5% are printed in bold.

In most cases a 10% change in the parameter barely influenced our measures. There are, however, certain measures that are particularly sensitive to changes in a specific parameter. MV, tMV, tMH and MT are sensitive to *A*, the parameter that sets the force that attracts the tip to its goal position. *A* is the parameter with which the movement time and thus the speed of the movement can be controlled. MGA mainly depends on *E*, the equilibrium distance of the virtual spring between the tips. When the stiffness of this spring (K) increases it causes a larger MGA earlier in the movement. The same was induced by a larger approach parameter in the model of Smeets and Brenner [3]. MH is mainly controlled by the repulsion from the table, *R_t_*.

Without changing the control principle used by the model it is thus possible to adapt the parameters in the model in order to make the movement satisfy different task requirements, such as making slower or faster movements or increasing or decreasing the safety margin, if there is a need to do so. However, the measures are clearly not excessively sensitive to the precise values of the parameters: a 10% change in the value of a parameter leads to a smaller percentage of change in all measures. In sum, if humans use a control mechanism similar to what we propose, prehension movements can be controlled by varying the parameters because kinematic variables are under the direct influence of the parameters but the model's general behavior does not change when the parameters are varied.
